# Elevated circulating levels of GFAP associated with reduced volumes in hippocampal subregions linked to mild cognitive impairment among community-dwelling elderly individuals

**DOI:** 10.3389/fnagi.2024.1461556

**Published:** 2024-10-29

**Authors:** Ying Zhang, Jun Wang, Haibo Zhang, Zhenkun Tan, Yingyan Zheng, Junjiao Ping, Jie Zhang, Jiali Luo, Linsen Li, Liming Lu, Xinxia Liu

**Affiliations:** ^1^School of Public Health, GuangDong Pharmaceutical University, Guangzhou, Guangdong, China; ^2^Research Laboratory, The Third People's Hospital of Zhongshan, Zhongshan, Guangdong, China; ^3^Department of Radiology, Zhongshan Torch Development Zone People's Hospital, Zhongshan, Guangdong, China; ^4^Department of Imaging, The Third People's Hospital of Zhongshan City, Zhongshan, Guangdong, China; ^5^Zhongshan Torch High-Tech Industrial Development Zone of Community Health Service Center, Zhongshan, Guangdong, China; ^6^Department of Psychiatry, The Third People's Hospital of Zhongshan City, Zhongshan, Guangdong, China; ^7^South China Research Center for Acupuncture and Moxibustion, Medical College of Acu-Moxi and Rehabilitation, Guangzhou University of Chinese Medicine, Guangzhou, Guangdong, China

**Keywords:** mild cognitive impairment, glial fibrillary acidic protein, hippocampus subfield, voxel-based morphometry, community

## Abstract

**Objective:**

Cerebrospinal fluid biomarkers are challenging to use for diagnosing mild cognitive impairment (MCI) in large populations, and there is an urgent need for new blood biomarkers. The aim of this study is to investigate whether astrocyte activation is correlated with hippocampal atrophy, and to assess the potential of glial fibrillary acidic protein (GFAP) as a biomarker for diagnosing MCI among community-dwelling older individuals.

**Methods:**

This cross-sectional study included 107 older adults. The levels of GFAP in serum were measured, and the volumetric assessment of gray matter within hippocampal subregions was conducted using Voxel-Based Morphometry (VBM). The relationship between hippocampal subregion volume and blood biomarkers were analyzed using partial correlation. The effectiveness of blood biomarkers in differentiating MCI was assessed using a receiver operating characteristic (ROC) curve.

**Results:**

We found that serum GFAP levels were significantly elevated in the MCI group compared to the cognitively normal (CN) group. Additionally, individuals with MCI exhibited a reduction gray matter volume in specific hippocampal subregions. Notably, the right dentate gyrus (DG) and right cornu ammonis (CA) subregions were found to be effective for distinguishing MCI patients from CN individuals. Serum levels of GFAP demonstrate a sensitivity of 65.9% and a specificity of 75.6% in differentiating patients with MCI from CN individuals.

**Conclusion:**

Specific atrophy within hippocampal subregions has been observed in the brains of community-dwelling elderly individuals. Elevated levels of circulating GFAP may serve as a sensitive peripheral biomarker indicative of hippocampal-specific cognitive alterations in patients with MCI.

## 1 Introduction

Mild cognitive impairment (MCI) represents a transitional phase between normal aging and Alzheimer's disease (AD) (Song et al., 2021), typically characterized by a subtle decline in memory, thinking, and learning capabilities, along with neurological alterations (Gaur et al., 2023). Long-term studies indicate that approximately 20% of individuals with MCI will eventually progress to AD (Qin et al., 2023). The etiology of the condition remains elusive, and the absence of effective treatment imposes a significant economic burden on patients' families and society at large. Therefore, early diagnosis and intervention during the initial stages of the disease, specifically during the MCI phase, are crucial in mitigating or even averting its progression.

The etiology of MCI hypotheses primarily encompasses the amyloid hypothesis, the tau protein hyperphosphorylation hypothesis, and the neuroinflammation hypothesis (Bradfield, 2023). Exploring the underlying mechanisms of these hypotheses is crucial for further understanding the pathology of MCI and for identifying biomarkers that can facilitate early diagnosis. Biomarkers, including CSF Aβ_42_/Aβ_40_, p-Tau181, t-Tau, and neurofilament light chains (NfL), can facilitate the early diagnosis of AD-related MCI and monitor disease progression (Chatterjee et al., 2023). Nevertheless, their high costs and invasive nature limit their utilization in large-scale population screenings, necessitating the search for novel, easily accessible biomarkers effective in diagnosing MCI.

In recent years, with the progress of bioassay technologies, such as immunomagnetic subtraction, single-molecule immunoarray, and immunoprecipitation-mass spectrometry (IP-MS), have intensified the search for peripheral biomarkers. Some biomarkers that are non-invasive, specific, sensitive, reproducible and easy to collect have become an important part of current AD screening and early diagnosis research (Ehrenberg et al., 2020). Recently, glial fibrillary acidic protein (GFAP) has garnered attention as a potentially valuable biomarker for various neurological disorders, including multiple sclerosis, frontotemporal dementia, AD, and Parkinson's disease. Furthermore, GFAP has been found to possess diagnostic significance in predicting the progression of MCI to AD (Cicognola et al., 2021; Abdelhak et al., 2022; Heimfarth et al., 2022). GFAP is distinguished as a specific marker for astrocytes in the central nervous system (CNS) and ranks among the cell-specific markers within the CNS (Kamphuis et al., 2014).

In addition to serving as a cytoskeletal element and a marker of mature astrocytes, GFAP is crucial for astrocytic differentiation and reactivity (Li et al., 2020). Neuroinflammatory processes, such as reactive astrocyte hyperplasia, may be linked to pathological mechanisms underlying neurodegenerative diseases (Osborn et al., 2016). Activated astrocytes are implicated in neuroinflammatory changes in AD through the release of cytokines, inflammatory factors, nitric oxide, and reactive oxygen species (Heneka et al., 2010). Furthermore, research indicates that activated astrocytes are involved in the degradation or clearance of Aβ, hinting at their potential as a novel therapeutic target in AD (Ries and Sastre, 2016). While most studies have focused on comparing control groups with disease groups, fewer have explored the various stages of AD. Blood GFAP levels have been identified as a biomarker for predicting the progression of MCI to AD (Cicognola et al., 2021). Plasma GFAP amplitude changes not only exceed those observed in cerebrospinal fluid but also more accurately distinguish between Aβ-positive and Aβ-negative individuals (Benedet et al., 2021). However, validation and exploration of related mechanisms across diverse ethnic populations remain limited.

Activated astrocytes can influence brain structure, including brain volume and white matter density (Takahashi et al., 2020). These structural changes can be quantitatively assessed using Voxel-based morphometry (VBM) technology. VBM is a neuroimaging technique that analyzes and evaluates structural changes in brain regions by segmenting and extracting the gray and white matter of the brain and detecting volume changes in these tissues (Huang et al., 2023). Brain atrophy is evident in patients with AD, with the most significant atrophy observed in the hippocampal region, which is crucial for memory formation (Ritchie et al., 2018). Functionally, the hippocampus holds a pivotal role in encoding and consolidating both short-term and long-term memories, as well as spatial processing. Structurally, the hippocampus comprises several anatomically and functionally diverse subfields, and analyzing these subregions allows for a more precise elucidation of their relevance to cognitive changes (Xiao et al., 2023). Segmentation of the hippocampus in postmortem AD brains reveals a robust correlation between atrophy of the cornu ammonis (CA)1 and subiculum regions and the diagnosis of AD (Blanken et al., 2017). Additional studies have demonstrated that the dentate gyrus (DG)/CA3 and subiculum regions at the lower end of the hippocampus exhibit more extensive and evident atrophy (de Flores et al., 2015). In this study, we aimed to compare the structural alterations in the hippocampus and its subregions between MCI patients and healthy individuals using VBM, while also exploring the hippocampal subregions that preferentially undergo astrocyte-responsive proliferation in MCI patients.

Consequently, the present study postulated that serum GFAP could function as a biomarker for MCI diagnosis and sought to evaluate its importance alongside other relevant blood biomarkers (specifically, t-Tau, p-Tau181, Aβ_42_, Aβ_40_, NfL) in differentiating healthy individuals from those suffering from MCI. By comparing the hippocampal subregion volumes between MCI patients and CN individuals, our objective was to identify hippocampal subregions associated with aforementioned blood biomarkers during the stage of MCI. This research serves as an exploratory foundation for elucidating the pathological mechanisms underlying MCI.

## 2 Methods

### 2.1 Research design and participants

In May 2023, a simple random sampling was employed to select one district in Zhongshan, Guangdong Province. A total of 500 community-dwelling older persons aged 60 and above were randomly selected to participate in the questionnaire survey. The inclusion criteria encompassed individuals aged 60 years and above who did not have any known major cerebrovascular diseases, dementia or other pathological cognitive impairments, acute functional mental disorders (including schizophrenia or bipolar disorder), stroke history and traumatic brain injury. Patients with a history of alcohol abuse, drug addiction, and long-term use of medications that affect cognitive function, such as glucocorticoids, antipsychotic drugs, and sedative-hypnotic drugs, are excluded.

Of the 91 people diagnosed with MCI, 52 agreed to participate in the study. A total of 55 participants were matched by sex, age, and education as a control group to the cognitively normal people in the same area. A total of 107 people were actively involved in the study by undergoing blood collection procedures. The APOE ε4 allele frequency was also investigated in participants who provided blood samples. Image data were collected within 7 days after completing the questionnaire. Nevertheless, due to non-cooperation from some participants and contraindications for image collection, MRI data was successfully collected from 86 individuals.

MCI was diagnosed according to guidelines issued by the National Institute on Aging and the Alzheimer's Association (Albert et al., 2011). The subject or their relative has expressed concern about the subject's cognitive abilities and an objective assessment indicated impairment in cognitive function, yet the subject can still maintain independent daily functioning and without dementia, then the subject could be diagnosed with MCI. In this study, we utilized the Mini-Mental Status Examination (MMSE) scale to evaluate cognitive function, the Activities of daily living (ADL) scale to assess daily living functions, and diagnosed dementia in accordance with DSM-5 criteria.

All volunteers provided written informed consent prior to participation, and the Zhongshan Third People's Hospital Ethics Committee, China (reference number SSYLL20220401) provided approval for the study.

### 2.2 Assessment of cognitive and abilities of daily living

Mini-Mental Status Examination (MMSE) (Winblad et al., 2004; Jia et al., 2021) consists of 30 items, that assess five dimensions: orientation, memory, attention and calculation, recall ability, and language ability. The correct answer gets 1 point, while the wrong answer gets 0 points, for a total of 30 points. A higher score indicates better cognitive function. MCI was identified using education-specific cutoff points for the total scores on the MMSE, which were categorized as 19 or less for illiterate individuals, 22 or less for those with an elementary school education, and 26 or less for individuals with a middle school education or higher.

Activities of daily living scale (ADL) (Wade and Collin, 1988) comprises 14 items, each scored from 1 to 4, with a total score ranging from 14 to 56. If an individual scored 3 or more on two or more items, or had a total score of 22 or higher, they were deemed to be functionally impaired and were excluded.

### 2.3 Blood sample acquisition and processing

All participants fasted overnight (not <8 h), and blood was collected in both plain and EDTA-treated 5 ml vacutainer tubes and gently inverted and mixed. After collection, the specimens were placed in universal transport boxes, stored at 4°C, and transported to laboratory within 4 h of collection. Plasma was separated from EDTA-treated blood samples and serum was separated from anticoagulant-free blood samples by centrifugation at 3,000 rpm for 10 min at 4°C.

Serum GFAP concentrations were determined by Single molecule immune, using a commercial reagent kit and an AST-Dx90 fully automatic fluorescence immunoassay analyzer from Su zhou Yu Jin Biotechnology. The brief steps were to mix and incubate the sample with magnetic beads indicating the presence of captured antibodies. The antigen in the sample bound to the captured antibody to form an antibody antigen complex. Detection antibodies labeled with fluorescent dyes were added to the antibody antigen complex, and combined with the antibody-antigen complex to form a double antibody sandwich structure. A washing solution was used to remove excess substances such as unbound fluorescent dyes. Finally, the fluorescence intensity of magnetic particles was detected, and the GFAP concentration in the serum of the sample was calculated by comparing the standard curve. The levels of Aβ_40_, Aβ_42_, NfL, p-Tau181, and t-Tau in plasma were measured using Simoa^®^ Neurology 3-Plex A Advantage Kit (N3PA, cat: 101995), Simoa^®^ NF-Light v2 Advantage Kit (cat: 104073), Simoa^®^ pTau-181 Advantage V2 Kit (cat: 103714), ALZpath Simoa^®^ p-Tau 217 v2 assay Kit (cat: 104371) in Simoa HD-X Analyzer ™ (Quante Rix Corp.). The coefficient of variation for GFAP was 47.77%, 33.93% for Aβ_40_, 31.83% for Aβ_42_, 18.13% for NfL, 52.32% for p-Tau181, 37.9% for t-Tau, respectively.

### 2.4 Brain imaging

#### 2.4.1 Image acquisition

All subjects underwent MRI scans using the same 3.0 Tesla Vida Siemens scanner (Siemens, Erlangen, Germany) with a 64-channel head coil. We used high-resolution 3D T1-weighted imaging sequences (3D magnetization prepared Fast Gradient Echo, MPRAGE). Scan parameters are as follows: repetition time (TR) = 2,000 ms, echo time (TE) = 2.26 ms, inversion time (TI) = 900 ms, flip angle, (FA) = 8°, field of view (FOV) = 256 × 256 mm, matrix = 256 × 256 mm, thickness = 1.0 mm, and voxel size = 1 × 1 × 1 mm^3^.

#### 2.4.2 Image processing

Voxel-based morphometry (VBM) analysis was performed using the CAT12 toolbox (https://dbm.neuro.uni-jena.de/vbm/, version 12.7, r1742) and the Statistical Parametric Mapping 12 (SPM12) (http://www.fil.ion.ucl.ac.uk/spm/software/spm12/) with default parameters in MATLAB version R2016b (The MathWorks, Inc., Natick, MA, USA). All scans were visually checked for artifacts and anatomical abnormalities by a senior neuroradiologist prior to pre-processing. Data quality was controlled by two methods: (i) all DICOM format images were reviewed before data processing to exclude images with artifacts or brain parenchymal lesions; and (ii) the weighted average image and preprocessing quality of all data was ensured to be at least B or above. Total intracranial volume (TIV) data were extracted.

During preprocessing, all 3D T1-weighted images were converted from DICOM format to Neuroimaging Informatics Technology Initiative (NIfTI) format. The default settings of the “Segment Data” module in CAT12 were employed, and all steps recommended in the manual were followed. The images were subsequently segmented into gray matter (GM), white matter (WM), and cerebrospinal fluid (CSF). The diffeomorphic anatomical registration through exponentiated lie algebra (DARTEL) algorithm was used for spatial registration, segmentation, and normalization of the images (Ashburner, 2007). DARTEL works by aligning gray and white matter in the image, creats a high-resolution average template, and registers all data to this template. The data were then normalized to a standard brain in the Montreal Neurological Institute (MNI) space. Using the MNI 152 standard space of the DARTEL template, the voxel size of the final standardized image was 1.5 × 1.5 × 1.5 mm. Spatial normalization corrects for differences in overall brain shape between subjects and removes non-brain tissue, thereby facilitating local adaptive segmentation. At the same time, these normalized images correct for signal intensity inhomogeneities. The segmented images are modulated by the Jacobian determinant of the transformation matrix to correct for volume changes.

After normalization, the resulting images were checked for data quality using the CAT12 “Check sample homogeneity” module, which revealed no potential outliers in brain volume. Subsequently, the segmented GM images were smoothed using an 8 mm full width at half maximum (FWHM) Gaussian kernel. Smoothing ensured the validity of the statistical analysis by making the data more normally distributed and reducing the individual differences among all subjects. To eliminate artifacts at the gray matter/white matter boundary, an absolute gray matter threshold of 0.1 was applied.

In this study, we will focus on selecting the hippocampus and its subregions as brain regions of subcortical interest. Based on previous literature (Jung et al., 2021), considering the complex shape and anatomical variation of the hippocampal region, we used the probability diagram of the cell structure of the hippocampal region to divide it into the following five subregions: cornu ammonias (CA), dentate gyrus (DG), entorhinal cortex (EC), subiculum (Subc), and hippocampal-amygdaloid transition area (HATA). In order to minimize the partial volume effect, the subfields were combined as follows: CA1/CA2/CA3 were combined as CA; CA4 and DG were combined as DG (Amunts et al., 2005). A total of 10 hippocampal subregion masks were obtained from the left and right sides, and the Gray Matter Volume of 10 regions of interest was extracted for comparison between groups.

### 2.5 Statistical analyses

Statistical analysis was performed with GraphPad Prism V.9.0 and IBM SPSS Statistics V.26.0. Continuous variables were described as unadjusted means with SD. Logarithmic was applied to the variables that were not normally distributed. Categorical variables were given as percentages (%). The *t*-tests and chi-squared tests were used to assess differences between the two groups.

The Analysis of Covariance (ANCOVA) was employed to compare the means of the detected parameters between the MCI and CN groups. We performed a voxel-based morphometric analysis using general linear models in SPM12, which included unrelated covariates such as age, gender, education, and total intracranial volume to reduce potential confounders. The mean volume of hippocampal gray matter and the mean volume of subregion gray matter were compared between the MCI and CN group. The familywise error (FWE) method was applied for multiple comparison correction, with significance established at *P* < 0.05 and a clustering threshold of *k* = 10. A partial correlation analysis was performed to assess the relationship between blood biomarkers and hippocampal subregion volume, controlling for age, sex, education, APOE ε4 carrier status and TIV as covariates. The diagnostic performance of biomarkers and hippocampal subregions was compared using receiver operating characteristic (ROC) curve analysis and the sensitivity and specificity refer to the values at the optimal cut-off point, as determined by maximizing the Youden's index. DeLong's test was utilized to compare the Area Under the Curve (AUC) for different models. A *P*-value of <0.05 was regarded as significant.

## 3 Results

### 3.1 Participant characteristics

As can be seen from Table 1, the characteristics of the two groups are evenly distributed. MCI patients were recruited at a mean age of 73.0 ± 7.4 years old, and 82.7% were women. MCI group and CN group were equivalent in age, gender, education, BMI, and total Intracranial volume.

**Table 1:** Characteristics of participants in MCI and CN groups (*n* = 107).

	**Entire (*n* = 107)**	**MCI (*n* = 52)**	**CN (*n* = 55)**	***t*/χ^2^**	***P***
**Demographics**					
Age	73.2 ± 7.1	73.0 ± 7.4	73.4 ± 6.9	0.045	0.791
Female, *N* (%)	81 (75.7)	43 (82.7)	38 (69.1)	2.688	0.101
Illiteracy (<1 years of education), *N* (%)	29 (27.1)	17 (32.7)	12 (21.8)	1.608	0.448
Primary school (1–6 years of education), *N* (%)	33 (30.8)	15 (28.8)	18 (32.7)		
Secondary school and above (>6 years of education), *N* (%)	45 (42.1)	20 (38.5)	25 (45.5)		
BMI [normal, *N* (%)]	51 (47.7)	25 (48.1)	26 (47.3)	4.587	0.101
BMI (overweight, *N* (%)]	35 (32.7)	13 (25.0)	22 (40.0)		
BMI (obesity, *N* (%)]	21 (19.6)	14 (26.9)	7 (12.7)		
**Others**					
APOE ε4 carrie, *N* (%)	4 (3.7)	1 (1.9)	3 (5.5)	–	0.618
TIV (mm^3^)	1,378.60 ± 140.40	1,348.06 ± 134.36	1,396.11 ± 128.99	1.215	0.228

CN, cognitively normal; MCI, mild cognitive impairment; BMI, body mass index; TIV, total Intracranial volume.

### 3.2 comparison of blood biomarkers between MCI and CN group

**Figure 1:**
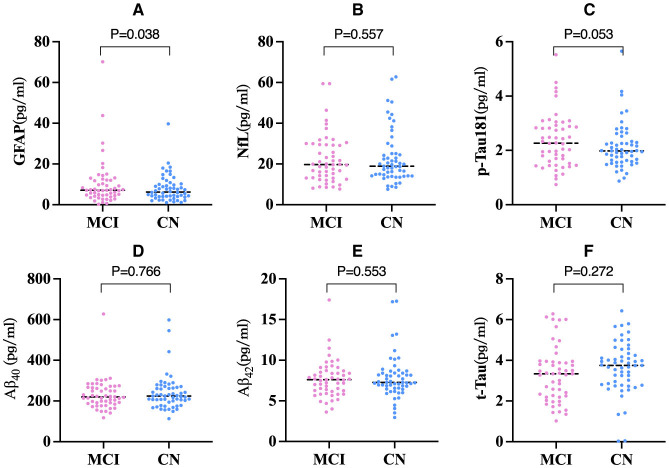
Comparison of blood biomarkers concentrations among two groups (*n* = 107). **(A)** Comparison of GFAP concentration among two groups. **(B)** Comparison of NfL concentration among two groups. **(C)** Comparison of p-Tau181 concentration among two groups. **(D)** Comparison of Aβ_40_ concentration among two groups. **(E)** Comparison of Aβ_42_ concentration among two groups. **(F)** Comparison of t-Tau concentration among two groups. *P*-values derived from ANCOVA with age, gender, education, and APOE ε4 carriers as covariates. ANCOVA, Analysis of covariance; The line segments depicted in the graph signify the median value of the data. GFAP, Glial Fibrillary Acidic Protein; NfL, Neurofilament Light; t-Tau, total Tau; p-Tau, phosphorylated tau; Aβ, amyloid-β.

GFAP levels were significantly higher in the MCI group than in the CN group after adjusting for potential risk factors (Li et al., 2020; Marizzoni et al., 2023) for AD such as age, gender, education, and APOE ε4 allele carrier status (*F* = 4.71, *P* = 0.038) (Figure 1A). However, no significant group differences were observed in the NfL, p-Tau181, Aβ_40_ and Aβ_42_, and t-Tau levels (all *P* > 0.05) (Figures 1B–F).

### 3.3 Comparison of hippocampal subregion gray matter volume between MCI and CN group

**Figure 2:**
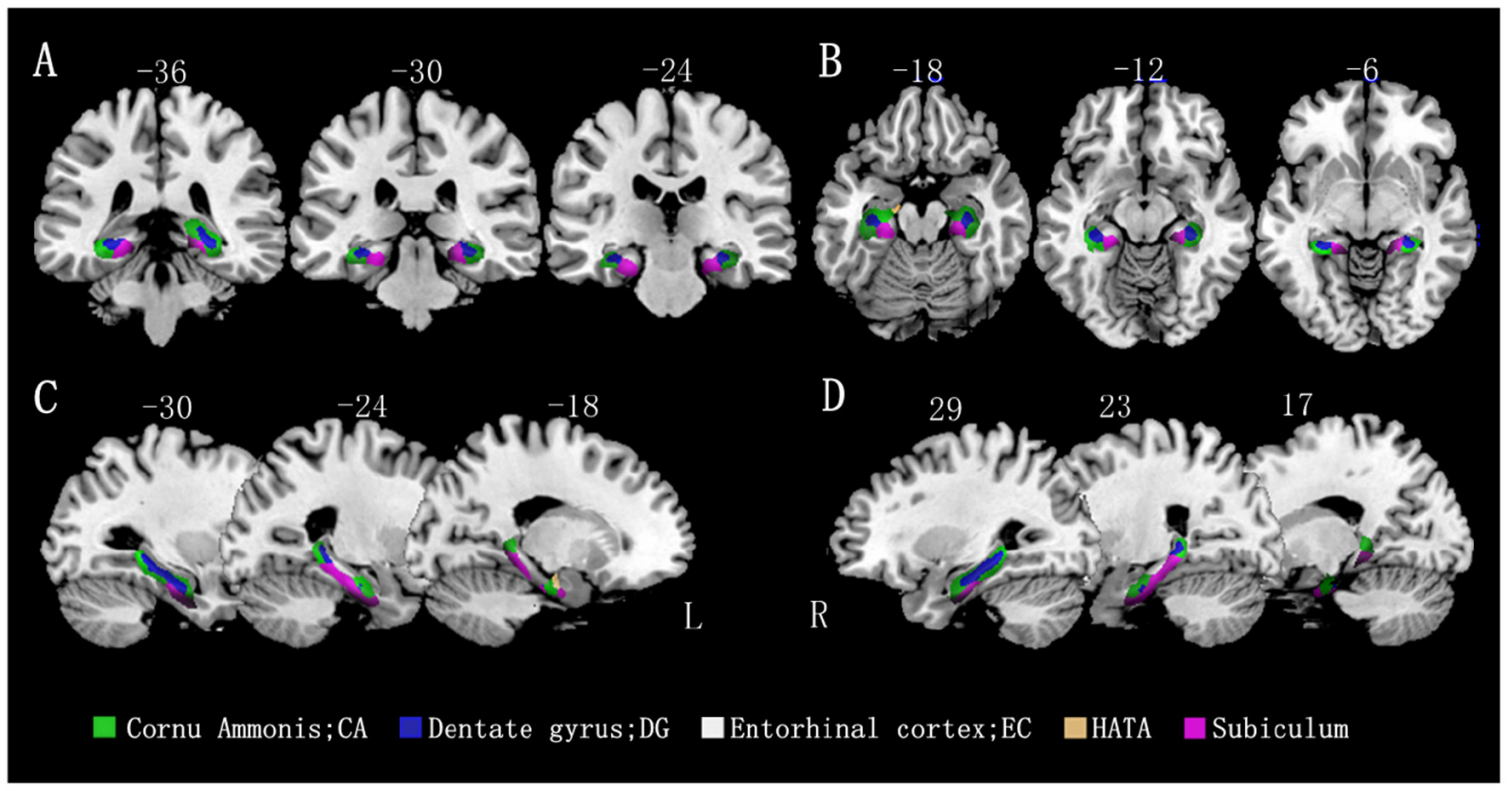
Differences in hippocampal subfield volumes between the two groups (*n* = 86). Different hippocampal subregions are represented by different colors. **(A)** Coronal view. **(B)** Transverse view. **(C, D)** Sagittal view. L, left; R, right.

A voxel-based morphometric analysis was performed using the general linear model in SPM12, with age, sex, education level, and total intracranial volume as covariates of no interest. The mean volumes of hippocampal gray matter and subregion gray matter were compared between the MCI group and control groups. The family-wise error (FWE) correction was applied to account for multiple comparisons. The results showed that all hippocampal subregions in the MCI group had lower volumes than those in the control group, with statistically significant differences observed in most subregions, except for the bilateral EC and rHATA. Please refer to Table 2 and Figure 2 for further details.

**Table 2:** Comparison of hippocampal subregion volume between MCI and CN groups (*n* = 86).

**Hippocampal subregion**	**Hemisphere**	**MCI group (*n* = 41)**	**CN group (*n* = 45)**	**Cluster size (voxels)**	***T*/*F* value**	***P*-value (FWE-cor)**	**MNI coordinates (mm)**		
							***x***	***y***	***z***
CA	Left	0.35 ± 0.05	0.38 ± 0.05	39	3.47	0.037	−28	−41	−5
DG		0.41 ± 0.06	0.44 ± 0.05	14	3.16	0.045	−29	−40	−4
lEC		0.43 ± 0.06	0.45 ± 0.05	186	2.04	0.310	−18	−7	−27
lHATA		0.34 ± 0.06	0.37 ± 0.05	23	2.49	0.049	−17	−17	−23
lSubc		0.37 ± 0.04	0.39 ± 0.03	13	3.28	0.044	−22	−33	−11
rCA	Right	0.38 ± 0.05	0.40 ± 0.05	93	3.71	0.032	26	−40	−3
rDG		0.41 ± 0.06	0.44 ± 0.05	261	3.66	0.030	27	−40	−3
rEC		0.42 ± 0.06	0.43 ± 0.07	N.A.	0.64	0.520	N.A.	N.A.	N.A.
rHATA		0.28 ± 0.05	0.29 ± 0.05	2	1.31	0.264	17	−10	−20
rSubc		0.37 ± 0.04	0.39 ± 0.04	28	3.55	0.042	23	−37	−6
lHIP	Left	3.40 ± 0.56	3.64 ± 0.46	N.A.	7.22	0.009	N.A.	N.A.	N.A.
rHIP	Right	3.27 ± 0.60	3.50 ± 0.49	N.A.	7.195	0.009	N.A.	N.A.	N.A.
TIV		1,348.06 ± 134.36	1,396.11 ± 128.99		0.007	0.934			

CA, cornu ammonis; DG, dentate gyrus; EC, entorhinal cortex; HATA, hippocampal-amygdaloid transition area; Subc, subiculum; HIP, hippocampus; TIV, Total Intracranial Volume; N.A., not applicable; MNI, Montreal Neurological Institute; *x, y*, and *z* correspond to the coordinates of the peak-activated voxel.

### 3.4 Discriminative power of hippocampal subfield volumes

**Figure 3:**
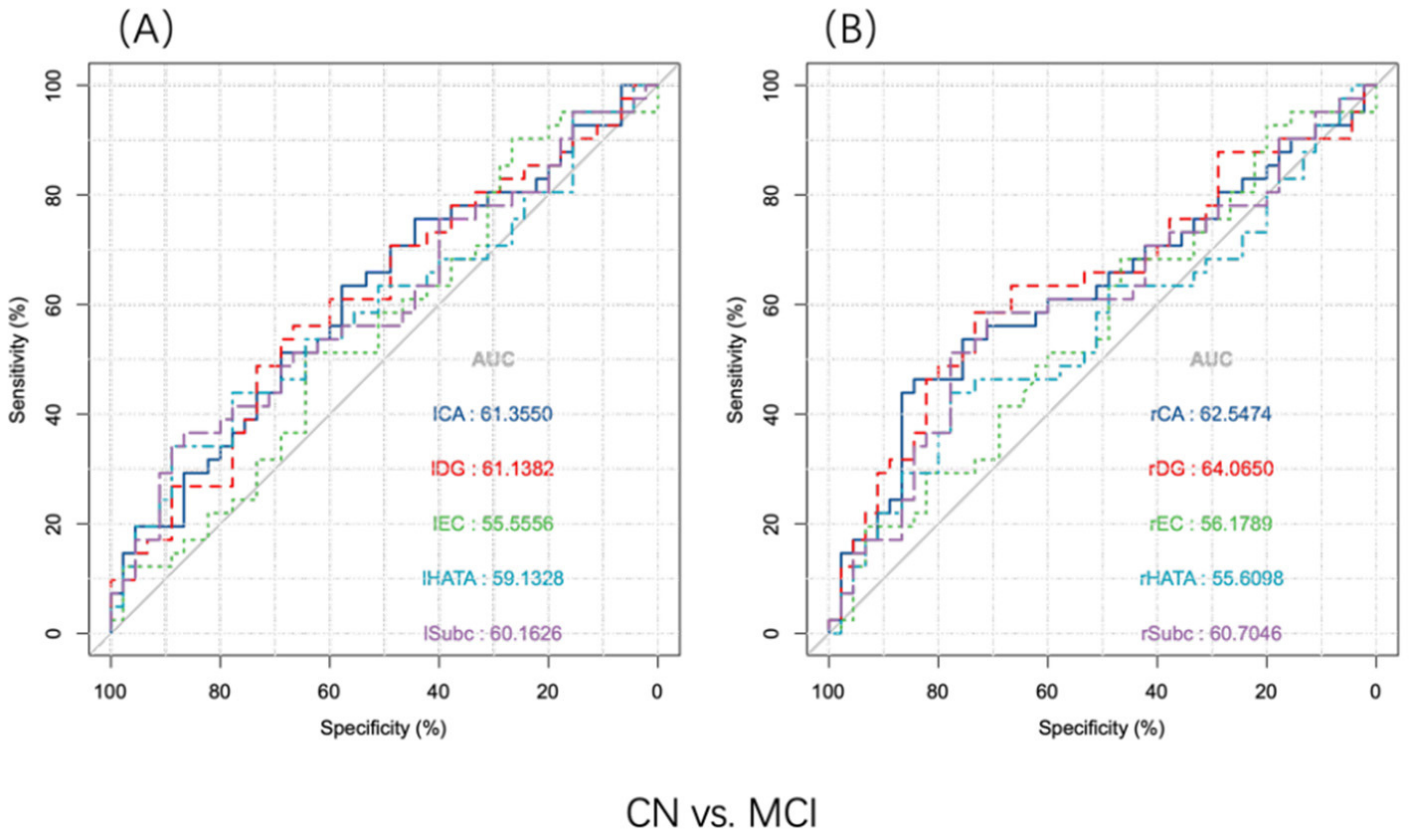
ROC analysis of the hippocampus subregion distinguished MCI and CN participants (*n* = 86). Discriminative power of hippocampal subfields volume. **(A)** Discernment of the volume of the left hippocampal subregion. **(B)** Discernment of the volume of the right hippocampal subregion. CA, cornu ammonis; DG, dentate gyrus; EC, entorhinal cortex; HATA, hippocampal-amygdaloid transition area; Subc, subiculum; l, left; r, right.

To evaluate the abillity of hippocampal subregions to distinguish between the two groups, ROC curves were analyzed separately for the left (A) and right (B) subregions (Figure 3). To differentiate between CN and MCI, all subfield volumes had similar AUCs (exceeding 0.556), but only the rCA and rDG subfields significantly distinguished between MCI and CN (*P* < 0.05) (Table 3).

**Table 3:** Detailed information of ROC analyses (*n* = 86).

**Hippocampal subfields**	**AUC**	**Sensitivity**	**Specificity**	***P***
lCA	0.614	0.634	0.578	0.07
lDG	0.611	0.561	0.667	0.076
lEC	0.556	0.902	0.267	0.375
lHATA	0.591	0.341	0.889	0.145
lSubc	0.602	0.366	0.867	0.105
rCA	0.625	0.463	0.537	**0.045**
rDG	0.641	0.585	0.733	**0.025**
rEC	0.562	0.683	0.317	0.326
rHATA	0.556	0.439	0.561	0.371
rSubc	0.607	0.585	0.415	0.088

The sensitivity and specificity refer to the sensitivity and specificity at the optimal cut-off point obtained by maximizing the Youden's index. ROC, receiver operating characteristic; AUC, area under the receiver-operating characteristic curve; CN, cognitively normal; MCI, mild cognitive impairment; CA, cornu ammonis; DG, dentate gyrus; EC, entorhinal cortex; HATA, hippocampal-amygdaloid transition area; Subc, subiculum; l, left; r, right. Bold represents *P* < 0.05.

### 3.5 Correlation analysis between hippocampal subregion volume and blood biomarkers

**Figure 4:**
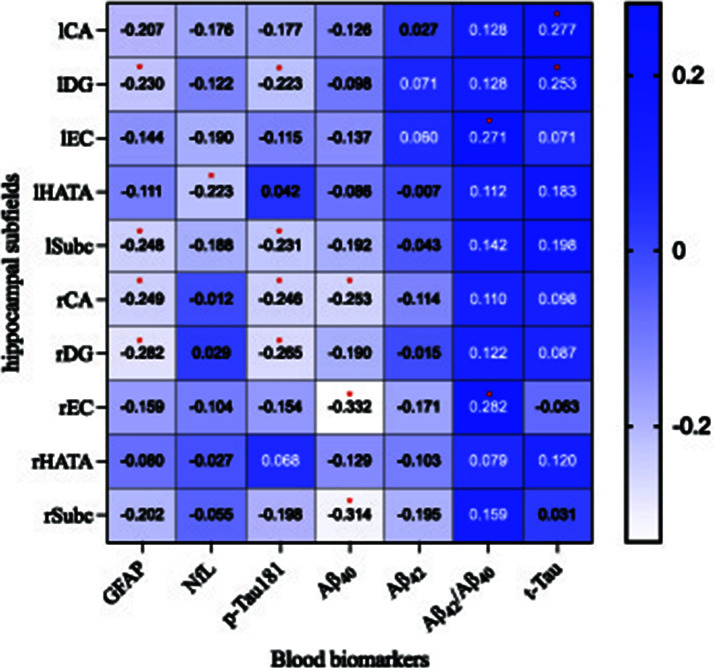
The partial correlation analysis illustrates the relationship between hippocampal subfield volumes and blood biomarker concentrations across all participants (*n* = 86). Here, blue indicates a positive correlation, with darker shades signifying stronger associations; conversely, white denotes a negative correlation, where a more intense hue corresponds to a more pronounced inverse relationship. *P < 0.05. CA, cornu ammonis; DG, dentate gyrus; EC, entorhinal cortex; HATA, hippocampal-amygdaloid transition area; Subc, subiculum; l, left; r, right. GFAP, glial fibrillary acidic protein; NfL, Neurofilament Light; t-Tau, total Tau; p-Tau, phosphorylated tau; Aβ, amyloid-β.

Figure 4 illustrates the partial correlations between hippocampal subregion volumes and blood biomarkers, adjusted for age, sex, APOE ε4 carrier status, and TIV. The findings revealed that GFAP levels were negatively associated with the volumes of the rDG (*r* = −0.282, *P* = 0.015), rCA (*r* = −0.249, *P* = 0.033), lSubc (*r* = −0.248, *P* = 0.021), and lDG (*r* = −0.230, *P* = 0.040). NfL levels showed a negatively association with the volume of the lHATA (*r* = −0.223, *P* = 0.046). Additionally, p-Tau181 levels were inversely related to the volumes of the rDG (*r* = −0.265, *P* = 0.018), rCA (*r* = −0.246, *P* = 0.028), lSubc (*r* = −0.231, *P* = 0.036), and lDG (*r* = −0.223, *P* = 0.045). Aβ_40_ levels were inversely related to the volumes of the rEC (*r* = −0.332, *P* = 0.004), rSubc (*r* = −0.314, *P* = 0.005), and rCA (*r* = −0.253, *P* = 0.020). The Aβ_42_/Aβ_40_ ratio was positively associated with the volume of the lEC (*r* = 0.271, *P* = 0.026) and rEC (*r* = 0.282, *P* = 0.018). Furthermore, t-Tau levels were positively associated with the volumes of the lCA (*r* = 0.277, *P* = 0.014) and lDG (*r* = 0.253, *P* = 0.024). No correlation was observed between Aβ_42_ levels and the volumes of any hippocampal subregions.

### 3.6 Receiver operating characteristic curves for the prediction of MCI vs. CN group

**Figure 5:**
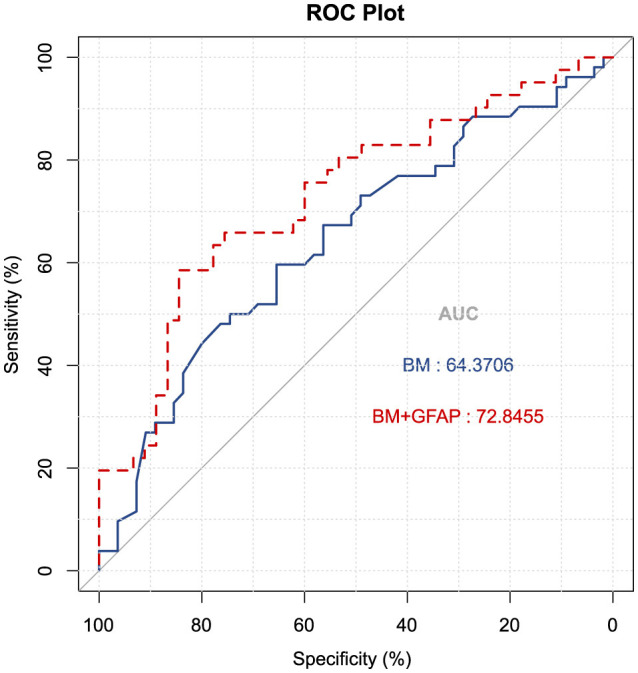
Receiver operating characteristic curves were utilized to predict MCI vs. CN (*n* = 107). The blue ROC curve represents the “base model” (BM), encompassing factors such as age, gender, education, and APOE ε4 allele status. The red ROC curve corresponds to the base model augmented with GFAP. AUC, area under the curve; GFAP, glial fibrillary acidic protein.

To evaluate the diagnostic efficacy of serum GFAP as potential biomarkers for differentiating MCI from CN individuals, logistic regression analyses were conducted with MCI or CN status as the dependent variable to compute predicted probability values, which were subsequently utilized to generate receiver operating characteristic (ROC) curves. A basic model (BM) was developed, incorporating established risk factors for AD, including age, gender, years of education, and the presence of the APOE ε4 allele; it yielded an AUC of 0.644 (*CI* 0.540–0.773), with a sensitivity of 31.1%, a specificity of 68.9% (*P* = 0.012). Upon integrating GFAP into the basic model, the AUC increased to 0.728 (*CI* 0.621–0.836), with a sensitivity of 65.9% and a specificity of 75.6% (*P* < 0.001). The DeLong's test indicated no significant difference between the two models (*Z* = −1.44, *P* = 0.149). See Figure 5 for details.

## 4 Discussion

This research demonstrates that the serum GFAP levels are higher in MCI patients from a community-based elderly cohort, suggesting that GFAP could potentially serve as a hematological marker for early detection of individuals predisposed to MCI prior to the onset of clinical symptoms. Despite the blood-brain barrier's role, which might distort the accuracy of blood biomarker levels as reflections of brain states, a meta-analysis by Gaur et al. (2023) supports these findings by indicating elevated GFAP in both cerebrospinal fluid and peripheral blood in MCI patients relative to a general cohort. Notably, the concentrations of GFAP in the subjects of this study were lower compared to the control populations in other studies. This variation may be attributed to our study's focus on community-dwelling elderly individuals, unlike other studies that primarily used samples from hospital settings or geriatric populations, where comorbidities might elevate GFAP levels. Additionally, elderly participants in such studies may present with multiple AD-related issues. Our participants, randomly selected seniors without self-reported symptoms, provide a baseline for possible widespread use of GFAP measurement. It is crucial to recognize that various factors, including demographics, behaviors, genetic predispositions, and others, could obscure the link between GFAP levels and MCI, with potential confounders impacting the results (Anderson, 2019; Gonzales et al., 2023). Variations in the testing methods also pose considerable influences. Furthermore, other biomarkers, such as Aβ_40_, Aβ_42_, NfL, t-Tau, and p-Tau181 were ineffective in distinguishing MCI patients from CN individuals. This ambiguity may arise from the MCI patients' relatively mild condition, resulting in lower biomarker levels in the blood. Additionally, the direct release of GFAP into the bloodstream from astrocytic end-feet at the neurovascular junction might mirror CNS alterations more closely than cerebrospinal fluid levels, underscoring the diagnostic utility of blood-based biomarkers (Pereira et al., 2021). In conclusion, increased GFAP levels in the blood suggest reactive astrocyte proliferation, indicative of early neurodegenerative processes. Therefore, GFAP holds promise as a biomarker to differentiate MCI from CN states.

The findings revealed that the MCI group exhibited a reduction in the volume of the bilateral DG, right CA, and left HATA when compared to the CN group. Furthermore, the volumes of the rCA and rDG subregions were effective in differentiating between individuals with MCI and those in the CN group. Multiple studies comparing MCI with hippocampal subregion volumes in a cognitively normal population are consistent with our results (Pluta et al., 2012; La Joie et al., 2013; Baek et al., 2022). Beak reported hippocampal CA4 and DG volume atrophy associated with increased Aβ deposition (Baek et al., 2022). Cognitive status was found to predict changes in hippocampal volumes, specifically in the CA1 and HATA regions (Becker et al., 2021). Functional magnetic resonance imaging (fMRI) studies conducted by Yassa detected hyperactive signals in the CA3 and DG regions of the hippocampus in aged rats, which were linked to deficits in spatial memory (Yassa et al., 2010). Such cognitive decline is typically reflected as atrophy in these hippocampal subregions, with the rCA and rDG displaying the strongest association coefficients. The recognized asymmetry in the bilateral hippocampal structure of mammals supports these observations (Hou et al., 2013). Disorientation a common symptom in AD and MCI, is associated with the volume of the right hippocampus (Peter et al., 2018). Studies in mice have found that signals from the CA3 of the right hemisphere connect to more robust synapses, playing a role in the stability of long-term memory maintenance (El-Gaby et al., 2015). The volumes of the right CA1 and DG in individuals with MCI are both smaller than those in healthy control groups (Qu et al., 2023). These studies provide compelling evidence for the significant atrophy in volume of the right DG and CA in MCI patients.

Additionally, our research revealed that elevated GFAP levels in the serum of patients with MCI were linked to diminished volumes in specific hippocampal subregions, including the bilateral DG, rCA, and lSubc compared to the control group. This correlation suggests that GFAP-associated pathologies may influence the atrophy of hippocampal subregions. Research by Cirarda corroborates our findings, demonstrating that GFAP levels correlate with volumetric changes in hippocampal subregions and are notably higher in the hippocampus of post-mortem patients (Díez-Cirarda et al., 2023). Neurodegenerative alterations in the CA1 region are linked to astrogliosis and reduced volume (Gonzalez-Rodriguez et al., 2021). However, the exact mechanisms behind the changes in hippocampal volume, whether due to the loss of neurons or glial cells, are still not fully understood. GFAP, a key protein in the astrocyte cytoskeleton, may have increased levels in the blood due to abnormal functional remodeling of astrocytes (Escartin et al., 2021). Microarray analysis also shows downregulation of the astrocytic cytoskeleton, indicating atrophy (Simpson et al., 2011). However, further research is needed to determine whether these changes indicate a neuroprotective response or whether they contribute to the exacerbation of neurodegeneration (Winblad et al., 2004). In summary, variations in the volumes of hippocampal subregions in MCI may reflect underlying changes in astrocytes, illuminating the complex relationship between astrocyte pathology and cognitive decline in MCI.

A ROC curve analysis was conducted to evaluate GFAP's ability to differentiate MCI from controls in this study. The combined diagnostic AUC for the baseline model and GFAP reached 0.728, demonstrating, a sensitivity of 65.9% and a specificity of 75.6%. Tao et al. (2024) reported a similar AUC of 0.714 for serum GFAP in distinguishing MCI from the control population, which aligns with our findings. Integrating GFAP with established AD risk factors significantly enhances the differentiation between MCI and control subjects, suggesting that GFAP levels are closely associated with age, sex, education, and APOE ε4 carrier status. However, predicting MCI based solely on serum GFAP is susceptible to various factors, necessitating the incorporation of multiple multidimensional factors for more accurate MCI prediction.

This study has several limitations. It was designed as a cross-sectional study, therefore, longitudinal tracking of patients could provide deeper insights into the pathophysiological changes associated with GFAP levels and cerebral alterations. Furthermore, we are unable to examine whether the associations of serum GFAP with demographic and clinical factors persist with adjustment for neuropathologic burden. Additionally, the small sample size may limit subgroup analyses, such as evaluating GFAP performance across different genders or lifestyles. Future research should involve larger cohorts to enhance the results' generalizability and applicability to a broader population.

In conclusion, this study demonstrates that GFAP is a promising biomarker for diagnosing MCI, exhibiting substantial sensitivity and specificity in distinguishing MCI from cognitively normal individuals in a community-based elderly cohort. MCI patients show reductions in hippocampal volume and elevated GFAP levels, potentially indicative of cognitive impairment. These findings may also guide future research toward identifying targets for new therapeutic interventions, including astrocyte-protective drugs, which could alleviate the adverse effects on hippocampal structures.

## Data Availability

The datasets presented in this study can be found in online repositories. The names of the repository/repositories and accession number(s) can be found in the article/Supplementary material.
