# Supplementary figures and images for: Elevated circulating levels of GFAP associated with reduced volumes in hippocampal subregions linked to mild cognitive impairment among community-dwelling elderly individuals
Source: Front Aging Neurosci. 2024 Oct 29;16:1461556. doi: 10.3389/fnagi.2024.1461556 (PMC11554497; doi:10.3389/fnagi.2024.1461556)

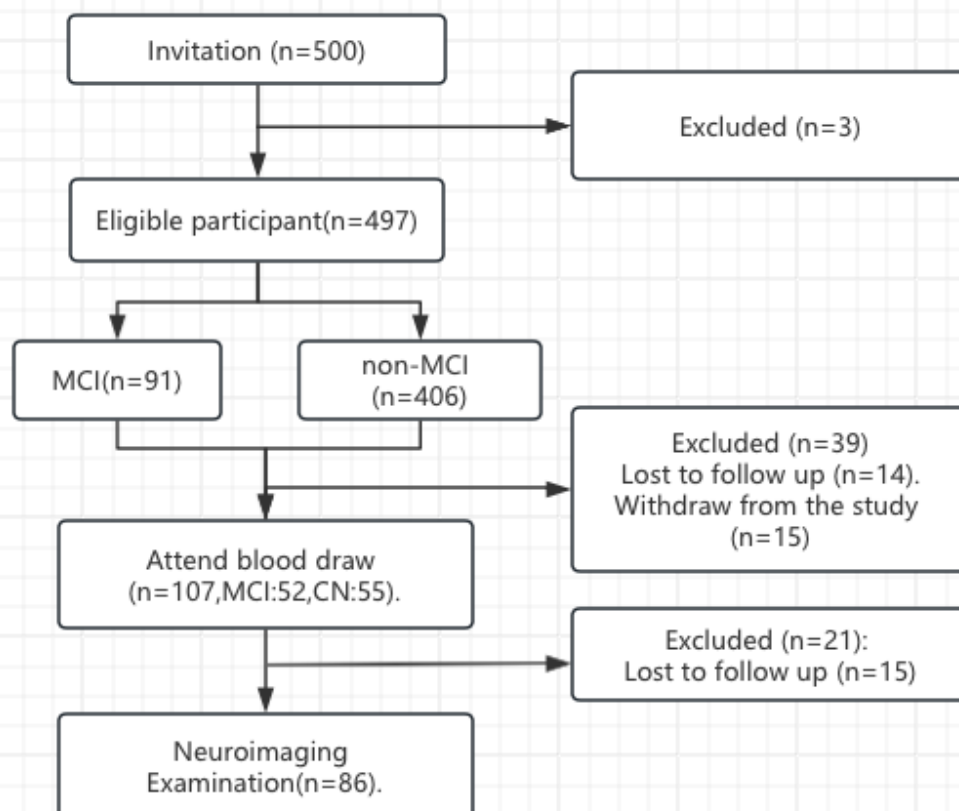

Supplement: Supplementary file 1 [file Data_Sheet_1.PDF]
